# Adolescent school absenteeism and service use in a population-based study

**DOI:** 10.1186/s12889-015-1978-9

**Published:** 2015-07-09

**Authors:** Kristin Gärtner Askeland, Siren Haugland, Kjell Morten Stormark, Tormod Bøe, Mari Hysing

**Affiliations:** Norwegian Institute of Public Health, Division of Mental Health, Department of Public Mental Health, Bergen, Norway; Regional Centre for Child and Youth Mental Health and Child Welfare, Uni Research Health, Bergen, Norway

**Keywords:** Adolescence, School absence, Service use, Truancy, School refusal, Youth@hordaland

## Abstract

**Background:**

School absenteeism is linked to a range of health concerns, health risk behaviors and school dropout. It is therefore important to evaluate the extent to which adolescents with absenteeism are in contact with health care and other services. The aim of the current study was to investigate service use of Norwegian adolescents with moderate and high absenteeism in comparison to students with lower rates of absence.

**Methods:**

The study employs data from a population-based study from 2012 targeting all pupils in upper secondary education in Hordaland County, Norway (the youth@hordaland-survey). A total of 8988 adolescents between the ages of 16 and 18 were included in the present study. Information on service use was based on adolescent self-report data collected in the youth@hordaland-survey. Absence data was collected using administrative data provided by the Hordaland County Council.

**Results:**

High absence (defined as being absent 15 % or more the past semester) was found among 10.1 % of the adolescents. Compared to their peers with low absence (less than 3 % absence the past semester), adolescents with high absence were more likely to be in contact with all the services studied, including mental health services (odds ratio (OR) 3.96), adolescent health clinics (OR 2.11) and their general practitioner (GP) (OR 1.94). Frequency of contact was higher among adolescents with moderate and high absence and there seems to be a gradient of service use corresponding to the level of absence. Still, 40 % of the adolescents with high absence had not been in contact with any services.

**Conclusions:**

Adolescents with high absence had increased use of services, although a group of youth at risk seems to be without such contact. This finding suggests a potential to address school absenteeism through systematic collaboration between schools and health personnel.

## Background

Absence from school is an important marker of functional impairment in adolescence as it is related to both current and future educational outcomes, as well as higher risk of unemployment [1, 2]. While a majority of adolescents are absent occasionally [3–5], as many as 14.3 % of Norwegian students in upper secondary school show chronic absenteeism defined as being absent more than 15 % of the school hours [4, 6]. The high rate of school absenteeism and drop-out has spurred the interest for early identification and intervention for adolescents at risk both in Norway and internationally.

The school is an obvious arena for interventions and a recent review showed that 75 % of indicated truancy interventions were school based [7]. Previous studies have found associations between absenteeism and a variety of health risk behaviors [3, 8–11], as well as somatic and mental health problems [12–14], suggesting health services as another possible arena. In keeping with this, adults who are absent from work are expected to see a doctor for identification of health factors, and collaboration between health personnel and the workplace is seen as important to reduce sickness certification and disability pensioning [15]. However, there is no systematic collaboration between the school and health sectors, and the extent to which adolescents with various levels of absence are in contact with these services is not known.

Previous studies suggest that there is a decrease in health service use from childhood to adolescence [16, 17] and adolescents are generally underrepresented in the health care system [16, 18, 19]. Academic functioning and school absence are among the factors related to help seeking in adolescence [16, 20], indicating that health services can be incorporated in interventions to reduce absenteeism.

The literature on school absence is characterized by various definitions, making it difficult to compare findings across studies. Today it is recognized that there is considerable overlap between constructs such as school refusal and truancy [21, 22] and there has been a call for studies using descriptive definitions of absenteeism [6, 23, 24]. This is especially relevant for studies of associations between service use and absenteeism, as there is expected to be heterogeneity in the group of youth with high absentee rates.

Based on the above considerations, the main goal of the present study was to assess the association between descriptive registry based absenteeism in upper secondary school and the use of school based services and health services. First, we wanted to explore the extent to which adolescents with low, moderate and high absenteeism are in contact with services, the frequency of such contact and the participants’ perception of causal factors. Further, we aimed to investigate differences in service use according to level of absence. Within this framework, it was an objective to identify characteristics of students with high absence never receiving services.

## Methods

### Study design

This population-based study employs previously collected data from the youth@hordaland-survey of adolescents in the county of Hordaland in Western Norway, conducted during spring in 2012. The youth@hordaland-survey is a cross sectional study with a main aim to assess mental health problems and service use in adolescents.

### Sample

All adolescents in the three age cohorts in Hordaland were invited to participate in the study (*n* = 19 430). The adolescents received information about the study and login details via their official school e-mail, followed by an SMS reminder for the majority of the students. One school class (about 45 min) during regular school hours was allocated for the completion of the Internet based questionnaire. A teacher was present to organize the data collection and to ensure confidentiality. For those not at school during the allocated school completion, the questionnaire could be completed at other times at their convenience during the study period. Some schools arranged catch up days, and we also arranged for participation for adolescents in hospitals or institutions during the study period. Those not enrolled in school at the time of the study received log on information through postal mail. However, adolescents who had dropped out of school were not included in the current study sample, as one of the main variables was school absenteeism.

Data from the youth@hordaland-survey include information on sociodemographic variables, familial socioeconomic status, use of health care and social services, daily life functioning, as well as extensive information on mental health. Of the 19 430 adolescents who were invited to participate, 10 220 (53 %) agreed to participate and 8988 (87.9 % of the original sample) approved the linkage to administrative data on school absence.

The study and the link between youth@hordaland and data on school absence were approved by the Regional Committee for Medical and Health Research Ethics in Western Norway.

### Instruments

#### Demographic information

Gender and year of birth are based on the personal identity number in the Norwegian national population registry. All participants were asked about their mother’s education, with the response options: ‘primary school’, ‘secondary school’, college or university: less than 4 years’ and ‘college or university: 4 years or more’.

#### Living situation

The participants’ living situation was based on self-report of a range of situations that were recoded as ‘living with family’, ‘living alone/with friends’, and ‘other’ for the present study. The variable ‘living with family’ includes living with biological parents, foster parents, adoptive parents, grandparents or another family. ‘Living alone/with friends’ includes living alone, living with friends or with a boyfriend/girlfriend.

#### School program

The educational programs reported by the participants were categorized into ‘general studies’, ‘vocational subjects in school’ (this categorization is based on the Norwegian high school system; including a program for general studies preparing for higher academic education, and a vocational education program), and a third option of ‘vocational training (work placement)’.

#### School absence

Administrative data on non-attendance were provided by Hordaland County Council. It included the number of days and school-hours each participant had been absent during the last semester (6 months), converted into percentage of absence relative to the total number of school days.

For the purpose of the present study high absence was defined as 15 % absence or more, based on Kearney’s criteria for problematic absence and the cut-off used in previous research on absenteeism [4, 6]. The participants were divided into three groups: Adolescents with low absence (less than 3 %), adolescents with moderate absence (between 3 and 15 %) and adolescents with high absence (15 % or more).

#### Self-reported absence

The participants were asked to report the number of days and school hours they had been absent during the past month. In addition, they reported location and behavior while absent, with the response alternatives: ‘I’m home’, ‘I’m out with friends’, ‘I’m at work’ or ‘I’m sick’. Other responses could be specified in an open field and multiple responses were also an option. The open responses were categorized into: ‘organizational work/politics/sport’, ‘unexcused absence’ and ‘other’.

#### Use of services

Service use was measured by the following question: “Have you had contact with the following services within the last school year? If yes, check how often”. The response categories used in the present study were; ‘school health services’, ‘special needs education’, ‘educational psychological service’, ‘mental health services for adolescents’, ‘mental health services for adults’, ‘general practitioner’, and ‘adolescent health clinic’. Additional services could be specified in an open field. In the present study the category ‘mental health services’ is a combination of mental health services for adolescents and adults. The participants who had been in contact with one or more services were asked to indicate the frequency of the contacts, measured by a Likert scale with the alternatives: ‘weekly’, ‘monthly’, ‘every three months’, ‘every six months’, and ‘less than every six months’, with the exception of ‘special needs education’. For the purpose of the present study, the latter two categories were combined in ‘every six months or less’ and ‘weekly’ and ‘monthly’ were combined in ‘monthly or more’.

### Statistics

In this study, we investigated service use in adolescents with low absence compared to adolescents with moderate and high absence. Service use was measured by numbers and category of services visited and frequency of contact. Chi-square tests were used to examine differences between adolescents with low, moderate and high absence with regards to demographic variables, rate of contact with specific services and self-reported absence. Differences in contact with each of the services studied were examined by logistic regression, using the absence variable as the exposure variable. Age, gender, maternal education and school program were included as control variables in the regression analyses. Multinomial logistic regression was used to calculate odds ratios for the number of services visited, ranging from ‘1’ to ‘4 and more’, and the frequency of contact for the participants according to absence. Results were considered significant at the *p* < .05 level. IBM SPSS version 21 for Windows was used for all analyses.

## Results

### Characteristics of the sample

The sample consisted of 8988 adolescents (51.5 % girls) between 16 and 18 years old in upper secondary education in the county of Hordaland, Norway. The majority of the participants were high school students in general studies (53.2 %) or vocational training at school (32.4 %). A majority lived with their family (90.2 %). Contact with services was common among the participants, 47.2 % of the sample had been in contact with one or more services the past semester.

### School absenteeism

A total of 910 participants (10.1 %) were absent 15 % or more of the school hours (labeled ‘high absence’) based on the school registry data. 4394 (48.9 %) of the participants were absent within the range of 3 and 15 % (labeled ‘moderate absence’) and 3689 (41.0 %) had less than 3 % absence (labeled ‘low absence’). There were more girls than boys in the group with high absence and a greater proportion of older adolescents with moderate and high absence compared to low absence (*p* < .000). More students in school based vocational training had high absence compared to participants in general studies or vocational training at the work place (*p* < .000). Living alone or with friends was more frequent among those with high absence (*p* < .000), and there was a tendency for adolescents with high absence to have less educated mothers (*p* < .000). For details on demographic information, see Table 1.

**Table 1:** Demographic variables in the youth@hordaland-survey (*n* = 8988)

	Low absence		Moderate absence		High absence		
	*n* = 3684		*n* = 4394		*n* = 910		
	41.0 %		48.9 %		10.1 %		
	%	n	%	n	%	n	*p*-value
Gender							<.000
Girls	36.6	1696	52.0	2407	11.3	525	
Boys	45.6	1988	45.6	1987	8.8	385	
Age							<.000
16	47.8	1729	45.2	1634	7.1	256	
17	41.7	1324	48.4	1536	9.9	314	
18	28.7	631	55.8	1224	15.5	340	
School program							<.000
General studies	39.5	1890	52.4	2506	8.0	383	
Vocational training (in school)	43.4	1262	44.3	1289	12.3	359	
Vocational training (work placement)	58.8	104	32.8	58	8.5	15	
Living situation							<.000
Living with family	41.5	3368	49.0	3976	9.4	765	
Living alone/with friends	36.7	280	46.2	352	17.1	130	
Maternal education							<.000
Primary school	34.4	245	50.8	362	14.9	106	
Secondary school (vocational training)	41.1	616	48.6	728	10.3	155	
Secondary school (general studies)	43.6	564	47.6	617	8.8	114	
College/university (<4 years)	42.6	551	49.5	640	7.9	102	
College/university (4+ years)	39.8	737	51.6	955	8.6	159	

*P*-value indicates significant differences between adolescents with low, moderate and high absence. The *p*-values are derived from chi-square tests

### Contact with services

**Fig. 1:**
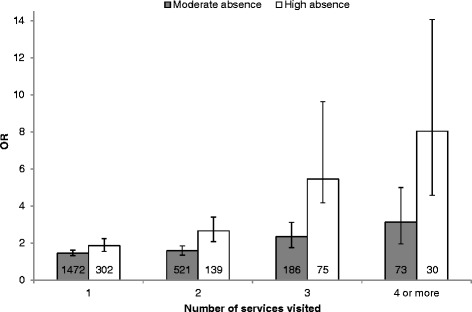
Odds ratios for contact with increasing number of services. Odds ratios and 95 % confidence intervals for adolescents with moderate and high compared to low absence. The number of participants in each group is presented in the bars

Among the adolescents with low absence, 40.6 % had been in contact with one or more services the past semester, compared to 53.8 % with moderate absence and 60.0 % with high absence (*χ*^2^(2, 8988) = 211.84, *p* < .000). There were statistically significant differences between adolescents with low absence compared to moderate and high absence with regards to the number of services they had been in contact with. Adolescents with moderate absence had an OR of 1.59 (95 % CI 1.35–1.86, seen in Fig. 1) for contact with two services, while the corresponding OR for adolescents with high absence was 2.67 (2.09–3.40). For contact with four or more services, the ORs were 3.13 (95 % CI 1.96–5.00) and 8.03 (4.58–14.06) for moderate and high absence respectively.

Among the adolescents with high absence, 40 % had not been in contact with any services. To investigate characteristics of these adolescents, the high absentee group was divided into students who reported contact with services (*n* = 546) and those who did not (*n* = 364). The only background variable that differed significantly between the groups was gender; there were more boys in the group that did not report contact with services (*p* < .000).

**Fig. 2:**
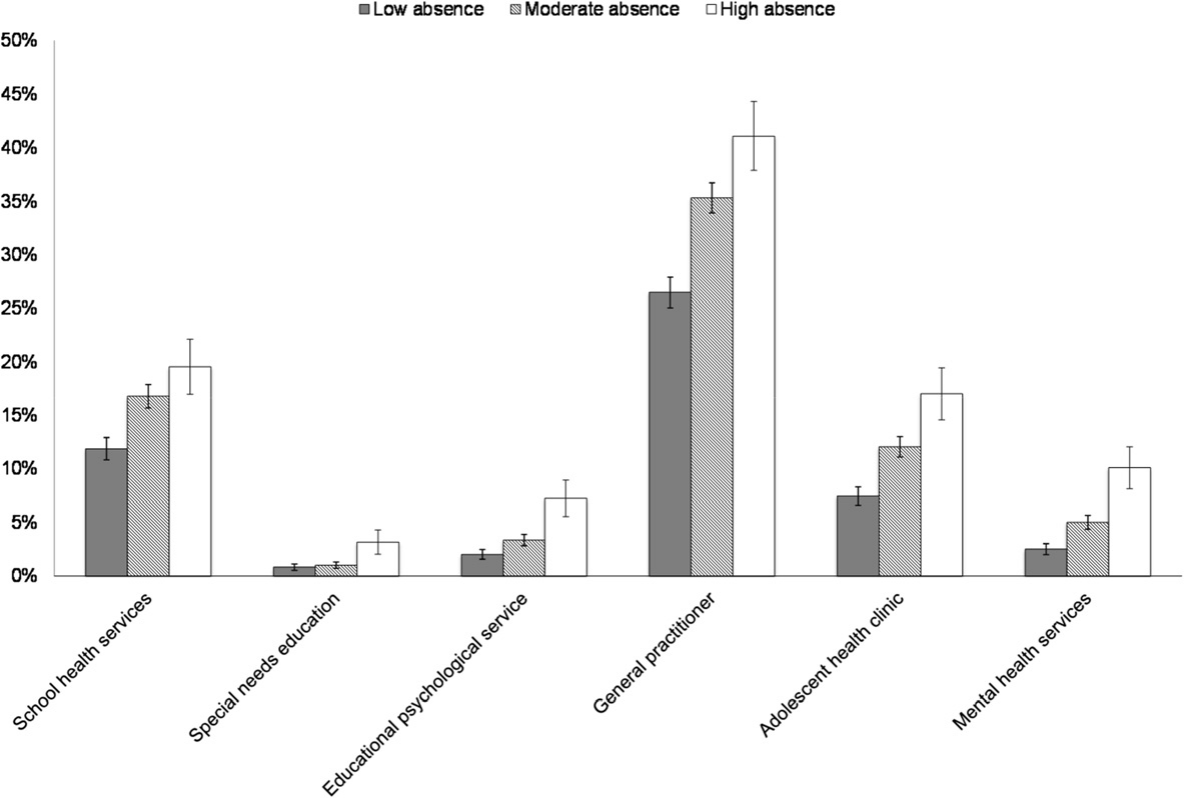
Percentage of participants in contact with specific services

Reports of service use was higher among adolescents with moderate and high absence compared to low absence for all services studied (see Fig. 2), with the exception of contact with special needs education where there was no difference between adolescents with low and moderate absence. For contact with the remaining services, there seems to be a gradient of service use from low to high absence, with the highest service use found among students with high absence. Although the absolute numbers were low, there was a twofold higher likelihood of mental health service use for adolescents with moderate absence (5.0 % versus 2.5 % for low absence) and a fourfold higher likelihood for those with high absence (10.1 % versus 2.5 %) compared to low absence (*p* < .000).

Adolescents with moderate and high absence were significantly more likely to have contact with the services studied compared to adolescents with low absence, with the exception of contact with special needs education for adolescents with moderate absence (see Table 2). Adjusting for age, gender, maternal education and school program reduced the estimates slightly, but the association between absence and service use remained. The statistically significant odds ratios range from 1.37 (school health services) to 2.03 (mental health service) for adolescents with moderate absence and from 1.68 (school health services) to 3.96 (mental health services) for adolescents with high absence.

**Table 2:** Odds ratios for contact with specific services

	Moderate absence		High absence	
	Crude	Adjusted*	Crude	Adjusted*
	OR (CI)	OR (CI)	OR (CI)	OR (CI)
Any service	**1.70 (1.56-1.87)**	**1.56 (1.42-1.72)**	**2.56 (2.20-2.99)**	**2.37 (2.01-2.79)**
School health services	**1.52 (1.34-1.73)**	**1.37 (1.20–1.56)**	**1.86 (1.53–2.25)**	**1.68 (1.38–2.06)**
Special needs education	1.23 (0.78–1.95)	1.36 (0.85–2.17)	**3.97 (2.38–6.62)**	**4.05 (2.37–6.91)**
Educational psychological service	**1.70 (1.28–2.25)**	**1.66 (1.25–2.21)**	**3.86 (2.75–5.42)**	**3.15 (2.22–4.47)**
General practitioner	**1.55 (1.41–1.71)**	**1.43 (1.30–1.58)**	**2.04 (1.75–2.38)**	**1.94 (1.65–2.28)**
Adolescent health clinic	**1.72 (1.48–2.01)**	**1.46 (1.24–1.71)**	**2.62 (2.12–3.25)**	**2.11 (1.68–2.65)**
Mental health services	**2.07 (1.62–2.65)**	**2.03 (1.58–2.60)**	**4.46 (3.31–6.01)**	**3.96 (2.91-5.39)**

Odds ratios and 95 % confidence intervals for adolescents with moderate and high compared to low absence

**Bold:** Statistically significant associations

*Adjusted for age, gender, maternal education and school program

### Frequency of contact

Adolescents with moderate and high absence were more likely to have frequent contact with all the services studied compared to those with low absence, with a tendency towards higher odds ratios for the more frequent contacts. The OR for contact with school health services every month was 2.86 compared to an OR of 1.32 for contact every three months for adolescents with moderate absence, as detailed in Table 3. Corresponding ORs for adolescents with high absence was 8.20 and 1.63, respectively. However, in the more extreme categories there are few participants, making the estimates unreliable as seen by the wide confidence intervals. Generally, adolescents with high absence were more likely to have frequent contacts with the services than adolescents with moderate absence.

**Table 3:** Odds ratios for frequency of contact

	Every 6 months		Every 3 months		Monthly	
	n	OR (CI)	n	OR (CI)	n	OR (CI)
**Moderate absence**						
*Crude*						
School health services	466	**1.30 (1.12–1.51)**	134	**1.78 (1.32–2.39)**	138	**2.36 (1.71–3.25)**
General practitioner	1117	**1.33 (1.20–1.47)**	303	**2.29 (1.85–2.84)**	131	**2.60 (1.86–3.63)**
Adolescent health clinic	377	**1.60 (1.34–1.91)**	110	**1.80 (1.29–2.50)**	43	**2.92 (1.57–5.44)**
Mental health services	57	1.49 (0.96–2.29)	19	1.36 (0.66–2.81)	144	**2.64 (1.89–3.68)**
Adjusted*						
School health services	466	1.17 (1.00–1.36)	134	**1.50 (1.11–2.03)**	138	**2.22 (1.60–3.08)**
General practitioner	1117	**1.24 (1.11–1.38)**	303	**2.05 (1.65–2.54)**	131	**2.47 (1.76–3.47)**
Adolescent health clinic	377	**1.32 (1.10–1.59)**	110	**1.55 (1.11–2.17)**	43	**2.86 (1.53–5.35)**
Mental health services	57	1.52 (0.99–2.35)	19	1.41 (0.68–2.93)	144	**2.52 (1.80–3.52)**
**High absence**						
*Crude*						
School health services	106	**1.48 (1.17–1.87)**	32	**2.12 (1.38–3.25)**	39	**3.32 (2.18–5.07)**
General practitioner	198	**1.24 (1.04–1.49)**	105	**4.19 (3.18–5.51)**	68	**7.12 (4.86–10.42)**
Adolescent health clinic	95	**2.06 (1.60–2.66)**	36	**3.01 (1.96–4.62)**	24	**8.34 (4.23–16.45)**
Mental health services	18	**2.39 (1.34–4.27)**	8	**2.92 (1.19–7.18)**	65	**6.07 (4.14–8.90)**
Adjusted*						
School health services	106	**1.30 (1.02–1.65)**	32	**1.71 (1.11–2.66)**	39	**3.18 (2.07–4.90)**
General practitioner	198	1.16 (0.97–1.40)	105	**3.69 (2.78–4.90)**	68	**6.82 (4.62–10.07)**
Adolescent health clinic	95	**1.63 (1.25–2.13)**	36	**2.48 (1.59–3.85)**	24	**8.20 (4.11–16.37)**
Mental health services	18	**2.45 (1.36–4.43)**	8	**2.82 (1.13–7.06)**	65	**5.76 (3.90–8.52)**

Odds ratios and 95 % confidence intervals for adolescents with moderate and high compared to low absence

**Bold:** Statistically significant associations

*Adjusted for age, gender, maternal education and school program

n = number of participants with the specified frequency of contact

### Self-reported absence

The participants who reported absence during the past term were asked to specify their location and behavior while absent. According to these self-reports, illness related absence was most common, reported by 28.0 % of the adolescents with low absence, 57.9 % of adolescents with moderate absence and 69.2 % with high absence (see Table 4). This was followed by staying at home (without illness), reported by 5.8 % of the participants with low absence, 16.5 % of those with moderate absence and 35.1 % with high absence. The participants with high absence reported significantly more absence for all reasons given (*p* < .000), except organizational work that was most common among those with moderate absence (*p* = .001).

**Table 4:** Self-reported reasons for absence

	Low absence		Moderate absence		High absence		
	*n* = 3684		*n* = 4394		*n* = 910		
	%	n	%	n	%	n	*p*-value
At home	5.8	214	16.5	724	35.1	319	<.000
With friends	0.5	19	1.5	66	3.7	34	.009
At work	0.2	8	0.9	38	1.6	15	<.000
Illness related	28.0	1031	57.9	2544	69.2	630	<.000
Organizational work/politics/sport	1.3	49	2.2	97	0.9	8	.001
Unexcused absence	3.5	128	5.4	238	6.5	59	<.000

*P*-value indicates significant differences between adolescents with low, moderate and high absence. The *p*-values are derived from chi-square tests

To examine differences among the high absentees, the group was divided into students with high absence who reported illness related absence (*n* = 630) and those who did not (*n* = 162). There were no significant differences regarding service use or differences in any of the background variables in the two groups.

## Discussion

The purpose of the present study was to examine service use among adolescents with different levels of absenteeism in a population based sample of Norwegian adolescents. Information on which services students with absence are in contact with is important to identify where students at risk can be met and where interventions can be implemented.

School absenteeism was frequent and the majority of the participants had been absent during the past semester. There was a greater likelihood of moderate and high levels of absence among girls, adolescents of low socioeconomic status, and those who were living alone or with peers. Among the adolescents with high absence, 40 % were not in contact with any of the studied health or school based services. However, a greater proportion of adolescents with moderate and high absence reported service use compared to participants with lower rates, and there seems to be a gradient of service use corresponding to the level of absenteeism. Adolescents in the moderate and high absence groups reported more frequent visits and were more often in contact with several services. The association between absenteeism and health service use was demonstrated across a wide range of services and persisted after controlling for possible confounders.

We found that 10.1 % of students had high absence (defined as being absent 15 % or more). This is somewhat lower than the 14.3 % that has been previously reported for Norwegian adolescents [4]. The discrepancy might be due to differences in data collection procedures, where the latter study increased the likelihood of responses from students with absenteeism by including two opportunities to complete the questionnaire at school. Although some of the schools participating in the present study arranged catch up days, this was not done consistently. Few studies report routine data on absence collected by the schools and comparison to other studies is therefore challenging. A study from the US showed lower rates of absence by assessing self-reported absence the past 30 days (11 % reporting any absence and 2 % reporting high absence) [25]. However, figures based on official records in the US estimate that between 14 to 37 % of students in the 12^th^ grade (17–18 years old) miss about 11 % of school or more [26]. Although not directly comparable, these figures indicate that high rates of absenteeism are common across countries.

Absence was more frequent among adolescents with less educated mothers, consistent with findings from previous studies [14]. Lower parental educational levels have been associated with a lower degree of parental academic involvement at home and in relation to school [27]. This can in turn increase the likelihood for truancy [28], possibly through lower academic achievement [29]. In the present study, a relatively high percentage of youths were not living with their family in the high absence group (17.1 %). These findings suggest that high school students living on their own may be in particular need of follow up to prevent absenteeism. While we have not assessed the causal factors, one possible hypothesis is that parents and caregivers have an important regulatory role in school behavior also in this age-group. Major conflicts with parents or problems in the family may also have contributed to relocation in late adolescence, thus identifying a group of vulnerable young people.

The heightened service use among adolescents with high absence in this study corresponds to previous studies [18, 20]. Interestingly, there seems to be a gradient in service use, with high absence being associated with the highest level of contact. A similar gradient was earlier found for the relationship between absenteeism and later employment and education [2]. Services organized in the primary health care (GPs, the school health services and the adolescent health clinic) were among the most visited, in line with studies that have demonstrated absenteeism as a predictor for primary health care attendance [20]. A total of 35.3 % of the adolescents with moderate absence and 41.1 % of those with high absence reported contact with their GP, which was the most visited service across absence levels. These findings suggest that primary health care services can be an arena for interventions, which is supported by a previous study showing that school-based health center use is associated with subsequent increase in attendance rates and grades [30]. Further, low to moderate use of such services has been associated with reductions in school drop-out, especially for high risk adolescents [31].

It is worth noting that 40 % of the adolescents in the high absence group had not been in contact with any of the services studied the past semester. This is in contrast to the follow-up of work absenteeism among adults, where systematic evaluation of health status, functional ability and the work environment is seen as crucial [15]. There may be a range of reasons for lack of contact, such as the young people’s reluctance to seek help [16] or a perception of absence as unrelated to health problems. Still, this cannot account for the lack of use of school based services which have a defined responsibility for school attendance.

### Strengths and limitations

Strengths of the study include a population based design with a large sample size and the use of administrative data on absence. The inclusion of frequency of contact strengthens the findings, and indicates that the association between absenteeism and services use is not merely a byproduct of one consultation the past semester. Including information about the health problems related to absence was beyond the scope of the present study. While we included a range of health services there may have been contact with other relevant services, e.g. counselors at schools.

The results should be interpreted in the light of some limitations. The cross-sectional design precludes conclusions regarding the causal directions of the results. In addition, contact with services during school hours could contribute to the association between absenteeism and service use, as shown in previous studies [20]. We only investigated mental health services within secondary health care, but we could expect higher rates of contact among adolescents with chronic illness [32]. This may have lowered the reported rates of contact. However, few somatic illnesses in this age group require high absence as defined here.

The sample might have been affected by methodological issues. Although all adolescents eligible for participation received the necessary information to complete the questionnaire at home, it is plausible that those attending school on the day of the study were more likely to participate. Absenteeism in the adolescent population is likely to be higher than reported in school based surveys, as described by Ingul et al [4] who found a non-significant tendency for higher absence among non-participants. Thus, the level of absence might be underestimated in the present study. There are currently no national or regional statistics of absence rates in Norwegian secondary schools available for comparison.

## Conclusions

School absenteeism is increasingly recognized as a public health problem, and has been suggested as an important point of intervention for the school system and health professionals [12]. Our results show that adolescents with high absence are frequent visitors to a range of services. However, we have identified a relatively large group of high absentees without any reported contact. In line with follow up of adults who are absent from work, there is a potential for increased and systematic evaluation of causal factors among youth. Absenteeism may also be examined as part of routine health care to identify unrecognized problems related to health, behavioral and social factors [33]. While there is still limited evidence for the effectiveness of interventions, a review of indicative prevention programs for chronic truant students suggests that they may be effective in increasing school attendance [7]. Reducing school absenteeism may be an important step in reducing disparities in educational achievement and negative outcomes. Consequently, there is a need for continued research on absenteeism and its correlates, as well as studies on the effect of interventions.

## Abbreviations

OR: Odds ratio
GP: General practitioner
